# Validation of $^{99m}$Tc and $^{177}$Lu quantification parameters for a Monte Carlo modelled gamma camera

**DOI:** 10.1186/s40658-023-00547-6

**Published:** 2023-04-08

**Authors:** Giovanni Di Domenico, Simona Di Biaso, Lorenzo Longo, Alessandro Turra, Eugenia Tonini, MariaConcetta Longo, Licia Uccelli, Mirco Bartolomei

**Affiliations:** 1 Department of Physics and Earth Science, University of Ferrara, via Saragat 1, 44122 Ferrara, IT Italy; 2 Medical Physics Unit, University Hospital, 44124 Ferrara, IT Italy; 3 San Bortolo Hospital, 44124 Vicenza, IT Italy; 4 Nuclear Medicine Unit, University Hospital, 44124 Ferrara, IT Italy; 5 Department of Translational Medicine, University of Ferrara, via Fossato di Mortara, 70 c/o viale Eliporto, 44124 Ferrara, IT Italy

**Keywords:** Quantitative imaging, Molecular radiotherapy, SIMIND Monte Carlo code, Quantitative activity estimation

## Abstract

**Purpose:**

Monte Carlo (MC) simulation in Nuclear Medicine is a powerful tool for modeling many physical phenomena which are difficult to track or measure directly. MC simulation in SPECT/CT imaging is particularly suitable for optimizing the quantification of activity in a patient, and, consequently, the absorbed dose to each organ. To do so, validating MC results with real data acquired with gamma camera is mandatory. The aim of this study was the validation of the calibration factor (CF) and the recovery coefficient (RC) obtained with SIMIND Monte Carlo code for modeling a Siemens Symbia Intevo Excel SPECT-CT gamma camera to ensure optimal $^{99m}$Tc and $^{177}$Lu SPECT quantification.

**Methods:**

Phantom experiments using $^{99m}$Tc and $^{177}$Lu have been performed to measure spatial resolution and sensitivity, as well as to evaluate the CF and RC from acquired data. The geometries used for 2D planar imaging were (1) Petri dish and (2) capillary source while for 3D volumetric imaging were (3) a uniform filled cylinder phantom and (4) a Jaszczack phantom with spheres of different volumes. The experimental results have been compared with the results obtained from Monte Carlo simulations performed in the same geometries.

**Results:**

Comparison shows good accordance between simulated and experimental data. The measured planar spatial resolution was 8.3$\pm 0.8$ mm for $^{99m}$Tc and 11.8±0.6 mm for $^{177}$Lu. The corresponding data obtained by SIMIND for $^{99m}$Tc was 7.8±0.1 mm, while for $^{177}$Lu was 12.4±0.4 mm. The CF was 110.1±5.5 cps/MBq for Technetium and 18.3±1.0 cps/MBq for Lutetium. The corresponding CF obtained by SIMIND for $^{99m}$Tc was 107.3±0.3 cps/MBq, while for $^{177}$Lu 20.4±0.7 cps/MBq. Moreover, a complete curve RCs vs Volume (ml) both for Technetium and Lutetium was determined to correct the PVE for all volumes of clinical interest. In none of the cases, a RC coefficient equal to 100 was found.

**Conclusions:**

The validation of quantification parameters shows that SIMIND can be used for simulating both gamma camera planar and SPECT images of Siemens Symbia Intevo using $^{99m}$Tc and $^{177}$Lu radionuclides for different medical purposes and treatments.

## Background

In modern nuclear medicine, the absolute quantification of SPECT images is fundamental for providing an estimate of the activity uptake in various organs and tissues for diagnostic assessments and therapeutic decisions. Monte Carlo (MC) simulation is a tool widely used to model real life systems, including nuclear medicine devices [1]. The MC method solves a problem numerically by using probability density functions (PDFs) and random sampling techniques. It starts from the description of particles’ interaction with matter to study the phenomena underlying the formation of images to optimise the data acquisition and processing steps. Due to the approximation and the simplification used in the description of physics laws inside a MC code, a mandatory step is the validation of MC model (code) before using it to simulate real world systems, in particular as a clinical simulator for SPECT imaging. To validate a MC code, outputs of simulated experiments are compared against results obtained from experimental measurements on the physical system. The validation ensures that the system performance matches that of the physical one in the range of parameters tested.

The absolute activity quantification consists of three steps: the first one is the reconstruction of the imaged volume from the projection images, compensating for photon attenuation, scatter and Collimator Detector Response (CDR). The attenuation is the principal factor reducing the number of photons hitting the gamma camera. Its value depends on the composition, density and thickness of tissue, together with the energy of gamma-ray photons passing through it. The scattered photons can be detected in the photopeak window, due to finite energy resolution of gamma-camera detection system, changing the number of detected photons under the photopeak. CDR is the degrading factor that affects the spatial resolution of SPECT images [2]. Several factors contribute to the CDR: photons that pass through the holes’ septa and photons which, despite the scattering with hole septa, have been detected. For measuring the scanner CDR one can use a capillary source placed at several source-to-detector distances while keeping the gamma camera head stationary [3]. Usually, the Gaussian + exponential function fits the measurements, and the fit results are used to model the distance-dependent CDR function [4]. The second step is to convert the reconstructed counts per second into activity [in MBq] through a Calibration Factor (CF). Different camera calibration methods have been proposed for evaluating CF: some researchers use planar scans of a small source [5] or of a Petri dish (following NEMA protocol for camera sensitivity test) [6], other ones use tomographic scans of a very simple phantom, such as a large cylindrical phantom (to avoid Partial Volume Effect, PVE), with a certain, known activity inside [7].

Planar acquisitions with a small or a planar source usually minimise the scatter and attenuation in the source; so, no corrections are needed for these degradation factors, and the CF can be obtained as the ratio between the decay corrected average counts per second and the source activity measured in MBq. Alternatively, the tomographic acquisitions mimic better the real acquisition performed on a patient, having similar scatter and attenuation, and additionally, the reconstructed 3D phantom volume includes the effects of the approximations used in the reconstruction of patient data. Nevertheless, the tomographic approach could be cumbersome when long half-life radioisotopes are used, especially considering radiation safety measures that should be in place. Due to a contaminated phantom. The CF unit is cps/MBq and it should be computed for every radionuclide and collimator used. The last step is to compute recovery coefficient (RC) factors in order to correct for the PVE: for small volumes, measured activity appears to be distributed over a volume larger than the true radioactive volume; this may lead to an underestimation of the activity in the real volume (and, then, of the measured absorbed dose) and to a wrong volume estimation. This is due to a blurring effect, caused by the finite spatial resolution of the gamma camera. PVE can be estimated through phantom studies, using the Jaszczak phantom with known volume spheres [7]. The RC coefficients are defined as the ratio of the measured activity concentration and the true activity concentration for each sphere, and they will be used to recover the true activity inside a small volume starting from the measured one.

Some authors have reported the comparison of measured and simulated gamma camera performances characteristics like spatial resolution, planar sensitivity, CF and RC for $^{99m}$Tc, $^{177}$Lu and $^{131}$I [8–11], both to investigate the influence a parameter has on systems’ performances and to validate the MC codes’ capability in modeling a specific gamma camera to use it later as a clinical simulator [12].

This study aims to validate the CF and the RC derived for a MC modelled gamma camera to ensure optimal SPECT quantification. Two objectives were set to achieve this aim. Firstly, the modelled gamma camera was verified by comparing the measured and simulated performance criteria: system resolution and sensitivity. Secondly, CF and RC were measured and compared with the simulated results.

## Materials and methods

This study was conducted in two parts: experimental data acquisition and Monte Carlo simulations. In each part both $^{99m}$Tc and $^{177}$Lu radioisotopes were studied, for a total of 40 experimental scans and 140 simulation runs. The information about the isotopes’ half-lives, their main gamma emissions and the maximum energy of their beta emission are summarized in Table 1.

**Table 1:** Decay characteristics of both Tc-99m and Lu-177; data from [25, 26]

Isotope	Half-life	Strongest $\gamma$ emission	Max $\beta$ energy	$\beta$ decay probability
		E$_\gamma$ [keV] (I$_\gamma$ [%])	E$_\mathrm{{max}}$ [keV]	I$_\beta$ [%])
Tc-99m	6.01 h	140.5 (88.5)	436.2	0.37
Lu-177	6.65 d	112.9 (6.2)	498.3	100
		208.4 (10.4)		

The SPECT/CT scanner used for the experimental measurements is a Siemens Symbia Intevo Excel [13] provided by Nuclear Medicine Unit, University Hospital of Ferrara (Italy). The system consists of two gamma camera detectors with NaI crystals (FOV 53.3x38.7 cm). The gamma camera parameters are listed in Table 2. The so-called “step and shoot” technique was used for the tomographic studies. The CT was performed after the SPECT acquisition, with a 110 kVp voltage and Care Dose 4D system, an automated exposure control which ensures constant image quality over all body regions at the lowest possible dose. The Symbia Intevo Excel was equipped with a Low Energy High Resolution (SY-LEHR) collimator for $^{99m}$Tc studies and with a Medium Energy Low Penetration collimator (SY-MELP) for $^{177}$Lu studies. All measurements and simulations were performed with a 20% energy window centred over the 140.5 keV photopeak of the $^{99m}$Tc, and with two 15% energy windows centred over the 113 keV and 208 keV photopeaks of the $^{177}$Lu. A Mec Murphil MP-DC-Chamber dose calibrator has been used for the activity measurement. The accuracy of the dose calibrator is better than 5% as stated from the last quality control test performed on it. All activity measurements were repeated five times to improve the statistical inaccuracy of the measurement. The $^{99m}$Tc radioisotope has been obtained as sodium perthecnetate ($Na[^{99m}Tc]O_4$) from $^{99}Mo/^{99m}Tc$ generator (Ultratechnekow, CURIUM, Netherlands), while the $^{177}$Lu has been obtained as Lutetium chloride ($[^{177}Lu]Cl_3$) (EndoLucinBeta, ITM, Munich, Germany).

**Table 2:** Main Symbia Intevo specifications, taken from Symbia T series data sheet.

Crystal size	59.1 x 44.5 cm$^2$
Crystal thickness	9.5 mm
PMT total number	59
PMT array	Hexagonal
System resolution at 10 cm, 140 keV	7.5 mm
Energy resolution at 140 keV	9.9%
Sensitivity at 10 cm, 140 keV	91 cps/MBq
SPECT reconstruction matrix size	128x128

The system parameters were measured with $^{99m}$Tc and LEHR collimator

### Phantom experiments for $^{99m}$Tc and $^{177}$Lu: planar imaging

Planar measurements aim at the evaluation of the fundamental SPECT features: spatial resolution and sensitivity, which are defined by the scintillation crystal, the collimator and the photodetector. The planar imaging procedures were performed according to the recommendations found in the report no.177 of AAPM “Acceptance Testing and Annual Physics Survey Recommendations for Gamma Camera, SPECT, and SPECT/CT Systems” [14]. All planar acquisitions were performed on a single detector only, as the gamma camera acceptance tests showed a slight difference between the system’s two detectors. All measurements have been repeated three times.

#### Extrinsic spatial resolution measurement

The system spatial resolution was measured using two capillary tubes with an inner diameter of 1 mm. The first tube was filled with 30 ± 1 MBq of a $^{99m}$Tc solution and placed on a low density support at a distance of 10.0 ± 0.5 cm from the collimator. Planar images were acquired in a 512x512 image matrix with a pixel size of 1.2x1.2 $mm^2$ until the highest pixel value in the line image exceeded 1000 counts.

A second capillary tube was filled with a 130 ± 7 MBq of a $^{177}$Lu solution, and planar images were acquired, as before. The spatial resolution was measured by drawing a horizontal profile across the image of the capillary tube in three different positions in order to compensate for the possible non-uniformity in the tube filling. The line profile was fitted with a Gaussian function and the full width at half maximum (FWHM) and the full width at tenth maximum (FWTM) values were calculated. The reference value provided by SIEMENS for the extrinsic spatial resolution with a LEHR collimator and a capillary tube filled with a $^{99m}$Tc source is 7.5 mm at 10 cm.

#### System sensitivity measurement

A Petri dish with an inner diameter of 10 cm was filled with 25.0 ± 1.3 MBq of a $^{99m}$Tc solution to a depth of 4 ± 1 mm. The dish was placed on low-density support made of polystyrene foam at a distance of 10.0 ± 0.5 cm from the collimator. Planar images were acquired in a 128x128 image matrix with a pixel size of 4.8x4.8 $mm^2$ until the total counts in the image exceeds 1 million. A background image was acquired for the same time after removing the radioactive source. A second Petri dish was filled with 30.0 ± 1.5 MBq of a $^{177}$Lu solution, and planar images were acquired, as before. The total net counts over the detector’s useful field of view (UFOV) was obtained and the sensitivity was calculated as follows,

1 $${\text{Sensitivity}}\;[{\text{cps}}/MBq] = \frac{{{\text{total}}\;{\mkern 1mu} {\text{net}}\;{\mkern 1mu} {\text{counts}}}}{{{\text{activity}}(MBq) \cdot {\text{acquisition}}{\mkern 1mu} \;{\text{time}}{\mkern 1mu} (s)}}{\text{ }}$$

The reference value provided by SIEMENS for the sensitivity with a LEHR collimator for $^{99m}$Tc source is 91.8 cps/MBq at 10 cm.

#### Dead time

Dead-time count loss may result in significant quantitation inaccuracy in SPECT imaging. In fact, at high count rates, the scintillation camera cannot be able to separate temporally all the incoming events, hence the count rate will decrease. This means that the gamma camera sensitivity is a diminishing function of the count rate. To estimate the dead-time effects on the gamma camera quantitation, additional measurements of the sensitivity have been performed for $^{99m}Tc$ by using values of activity between 4 MBq to 3500 MBq. The TEW scatter correction were applied to the measured photopeak counts to remove the scatter or pile-up events could accumulate under the photopeak [15].

### Phantom experiments for $^{99m}$Tc and $^{177}$Lu: tomographic imaging

#### Calibration factor measurement

**Fig. 1:**
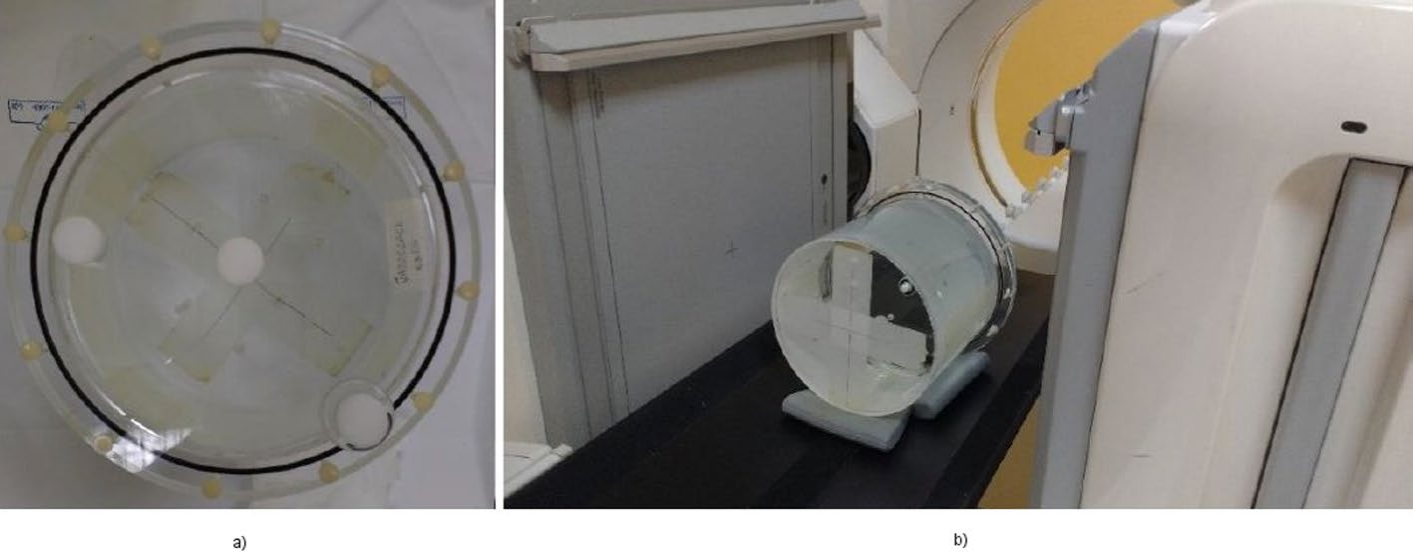
Experimental Setup Example of experimental configuration: **a** the uniform phantom is shown; **b** the acquisition geometry for Tc-99 m

A cylindrical Jaszczak SPECT Phantom (Fig. 1) deprived of all inner inserts has been employed to obtain the CF. The cylinder was filled with a 6800 ml solution of distilled water, 350 MBq of $^{99m}$Tc.

Similarly, the Jaszczak phantom was filled with a 6800 ml solution of distilled water (6720 ml), 430 MBq of $^{177}$Lu from a certificated vial (accuracy of ±10%), and 67 ml of HCl (37%). The hydrochloric acid was added to prevent lutetium accumulation on phantom surfaces and to ensure a homogeneous radioactive solution.

For both $^{99m}$Tc and $^{177}$Lu, the SPECT/CT acquisitions were performed via the Siemens Symbia Intevo Excel with the step-and-shoot technique. Each tomographic acquisition consisted of 64 projections over 360$^{\circ }$ projections performed maintaining a constant distance of 25 cm between the center of the cylinder and the lower part of the detector head. The acquisition time was 20 s for $^{99m}$Tc and 30 s for $^{177}$Lu respectively.

The reconstruction of the projected images was performed with the built-in software from the vendor, Siemens Flash-3D, based on the OSEM-3D iterative reconstruction technique [16]; employing 10 iterations and 8 subsets were chosen. CT-based attenuation-, window-based scatter- and CDR corrections were applied during the reconstruction process. Flash-3D models CDR in both transverse and axial directions.

The scatter correction for Technetium was performed via the DEW (Double Energy Windows) technique with the use of the PW (Photopeak Window) and the LSW (Lower Scatter Window).

The scatter correction for Lutetium was performed via the TEW (Triple Energy Windows) method for the 113 keV peak and the DEW method for 208 keV peak; the widths of each photopeak window are reported in Table 3. A Gaussian post-reconstruction filter with 4.8 mm FWHM was applied to the reconstructed volume.

**Table 3:** Photopeak Window (PW), Low Scatter Window (LSW), Upper Scatter Window (USW) for $^{99m}$Tc and $^{177}$Lu main peaks are listed

Radionuclide	Main peak [keV]	PW range [keV]	LSW range [keV]	USW range [keV]
Tc-99m	140.5	123.9–151.4	103.2–123.9	not used
Lu-177	113	102.7–119.4	86.1–102.7	119.4–128.2
Lu-177	208	189.3–220.0	168.8–189.3	Not used

#### Recovery coefficients measurement

To obtain the RC coefficients, the absolute quantification of $^{99m}$Tc was performed via a Jaszczak SPECT Phantom with six hot spheres.

The phantom was placed in the centre of the field of view. Acquisitions were performed with the same settings as those of the uniformly filled Jaszczack phantom previously described and conducted with 64 projections of 20 s scan time.

The energy windows were the same as those set for the CF evaluation and are listed in Table 3. The spheres’ volume and the background activity are listed in Table 4. Each activity value reported in Table 4 is the mean of five different measurements.

**Table 4:** The six spheres of the Jaszczack phantom with their respective activity and the background are shown. Each of the value reported in this Table is the mean value of five different measurements, with a standard deviation less than 1%. These errors must be added to the 10% error on the activity as stated by the dose calibrator manufacturer

Sphere volume [ml]	Sphere diameter [mm]	$^{99m}$Tc activity [MBq]
0.5	9.8	4.8
1.0	12.4	4.4
2.0	15.6	4.5
4.0	18.9	4.7
8.0	24.8	4.7
16.0	31.2	4.6
Phantom Volume [ml]		Background Activity [MBq]
6800		131.7

For the evaluation of the RC coefficients of $^{177}$Lu a NEMA image quality PET phantom with five spheres of different diameters was used. Spheres diameters, volumes, injected and background activities are listed in Table 5. Measurement settings were the same as those used for the CF evaluation and are listed in Table 2 and Table 3. The number of projections was 64, each of 30 s duration.

**Table 5:** The five spheres of the NEMA PET phantom with their respective activity and background are shown. Each of the value reported in this Table is the mean value of five different measurements, with an associated error of less than 1%

Sphere volume [ml]	Sphere diameter [mm]	$^{177}$Lu activity [MBq]
1.2	13.0	8.3
2.6	17.0	8.4
5.6	22.0	8.6
11.5	28.0	8.7
26.5	37.0	7.5
Phantom Volume [ml]		Background Activity [MBq]
9600		201.0

The ratio between activity concentration in the background and the activity concentration in the spheres was not constant but ranged from 0.2% to 7% starting from the smallest sphere to the largest. Each value reported in Table 5 is the mean of five different measurements, with an associated standard deviation of less than 1%.

CT data has been used for delineation of the volume of interest (VOI) of each sphere in SPECT studies.

The curve fitting the RC values was performed using the Igor software [Igor Pro, version 4.01, Wavemetrics, Inc, 1988-2000, Oregon, USA]. RC data errors were evaluated by taking into account the Poisson distribution of the SPECT acquired counts and the errors in activity measurement, volume and time interval estimation.

### Monte Carlo simulation for $^{99m}$Tc and $^{177}$Lu

Monte Carlo simulations of the experiments performed with $^{99m}$Tc and $^{177}$Lu have been performed via SIMIND v6.1. The Monte Carlo simulation code SIMIND is a photon-tracking program developed by Professor Michael Ljungberg (Medical Radiation Physics, Department of Clinical Sciences, Lund, Lund University, Sweden). SIMIND models a standard clinical SPECT camera, then simulates projection images from user-defined attenuation maps and activity distributions.

Both $^{99m}$Tc and $^{177}$Lu were studied via SIMIND: the main parameters set for the Monte Carlo simulations are listed in Table 6.

**Table 6:** Main parameters inserted in SIMIND’s CHANGE program for horizontal cylinder uniformly filled with radionuclides activity

	$^{99m}$Tc	$^{177}$Lu
Photon energy	140 keV	113 keV and 208 keV
Source type	Horizontal cylinder	Horizontal cylinder
Energy resolution	9.9% @140.5keV	9.9% @140.5keV
Intrinsic Resolution	0.38 mm	0.38 mm
Photons per projection	$10^7$	$10^7$
Distance to detector (circular orbit)	25 cm	25 cm
Matrix Size	128x128	128x128
Acceptance angle	45$^{\circ }$	45$^{\circ }$
Rotation mode	CW	CW
Rotation angle step	5.625$^{\circ }$	5.625$^{\circ }$
Number of projection	64	64
Collimator	Sy-LEHR	Sy-ME

In SIMIND, we simulated all the planar and tomographic acquisitions reported previously, and we added the simulation of the system spatial resolution for distances from the source to collimator front-end ranging from 5 cm to 40 cm in 5 cm steps both for $^{99m}$Tc and $^{177}$Lu. These curves are useful to estimate the compensation for system spatial resolution in the reconstruction process. To obtain the three-dimensional studies, the projected images produced via SIMIND were reconstructed using CASToR (Customizable and Advanced Software for Tomographic Reconstruction [17]), an open-source toolkit for tomographic reconstruction for both emission and transmission exams. CASToR applies the OSEM-3D iterative reconstruction technique [18], 10 iterations and 8 subsets were chosen. Attenuation correction was performed using the SIMIND generated density maps, including window-based scatter correction, while the CDR was modelled as a stationary 2D isotropic Gaussian.

## Results

### Planar system spatial resolution

The measured and simulated planar system spatial resolution, stated as FWHM and FWTM, at a source-detector distance of 10 cm are reported in table 7 both for $^{99m}$Tc and $^{177}$Lu.

**Table 7:** Comparison of measured planar System Spatial resolution with Monte Carlo results.

Radioisotope	Main peak [keV]	FWHM[mm]		FWTM[mm]	
		Measured	Simulated	Measured	Simulated
Tc-99m	140.5	8.3 ± 0.8	7.8 ± 0.2	14.9 ± 0.9	14.2 ± 0.2
Lu-177	113.0	11.7 ± 0.4	11.3 ± 0.6	21.3 ± 0.7	20.6 ± 1.0
Lu-177	208.0	11.8 ± 0.6	12.4 ± 0.4	21.6 ± 1.2	22.0 ± 0.7

All parameters have been measured at distance of 10 cm from collimator

**Fig. 2:**
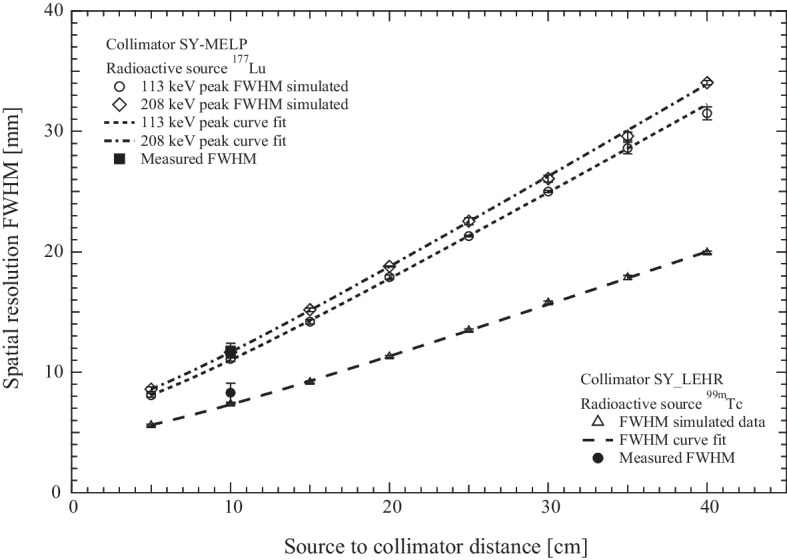
Simulated Spatial resolution for $^{{99m}}$Tc and $^{{177}}$Lu. Plot of spatial resolution as function of distance between source and detector for $^{99m}$Tc and $^{177}$Lu

In Fig. 2a it is shown the simulated spatial resolution for $^{99m}$Tc as function of source to detector distance D, while in Fig. 2b the spatial resolution for the two peaks of $^{177}$Lu are plotted. The simulated data are fitted with the curve suggested by Frey et al. [4],

2 $${\text{FWHM}} = \sqrt {(a \cdot D + b)^{2} + c^{2} }$$

and the value of $\chi ^2_r$ is 0.72 for $^{99m}$Tc, while the $\chi ^2_r$ values for the 113 keV and 208 keV peaks of $^{177}$Lu are 0.8 and 0.64 respectively.

### Planar system sensitivity

In table 8, the measured and the simulated sensitivity at a source-detector distance of 10 cm both for $^{99m}$Tc and $^{177}$Lu are reported. The experimental results agree well with the SIMIND outcomes, apart from the 208 keV sensitivity: the experimental values are nearly 13.6% lower than that obtained with SIMIND.

**Table 8:** Comparison of measured planar System Sensitivity with Monte Carlo results. All parameters have been measured at distance of 10 cm from collimator

Radioisotope	Main peak [keV]	Sensitivity [cps/MBq]	
		Experimental	Simulated
Tc-99m	140.5	88.0 ± 4.4	89.4 ± 0.5
Lu-177	113.0	9.9 ± 0.5	9.7 ± 0.1
Lu-177	208.0	9.6 ± 0.5	10.9 ± 0.2

### Dead time

**Fig. 3:**
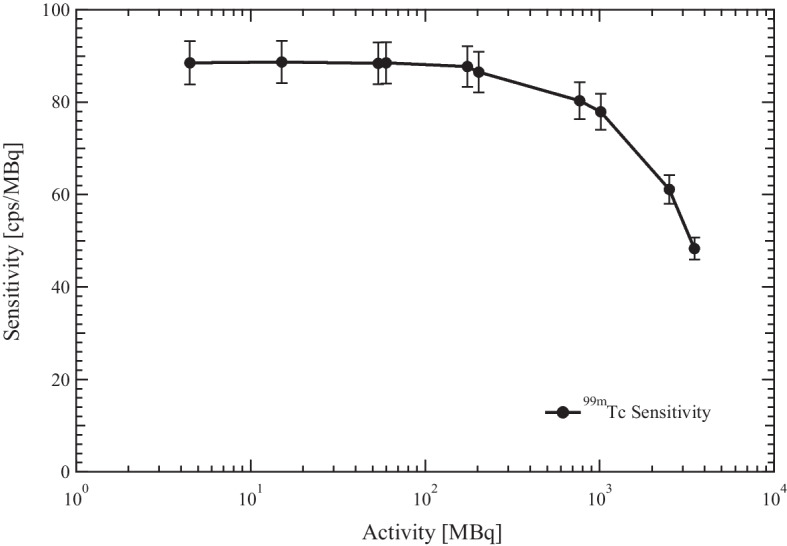
Planar Sensitivity of $^{{99m}}{Tc}$ as a function of activity. Plots of $^{99m}Tc$ planar sensitivity as a function of activity

Figure 3 shows the additional measurements of $^{99m}Tc$ planar sensitivity as function of activity. The sensitivity value is 88.6 ± 4.5 cps/MBq for the $^{99m}Tc$ activity below 200 MBq, above this activity level the sensitivity decrease until to reach the value of 48.3 cps/Mbq for the activity of 3500 MBq.

### CF and RC for $^{99m}$Tc and $^{177}$Lu

**Fig. 4:**
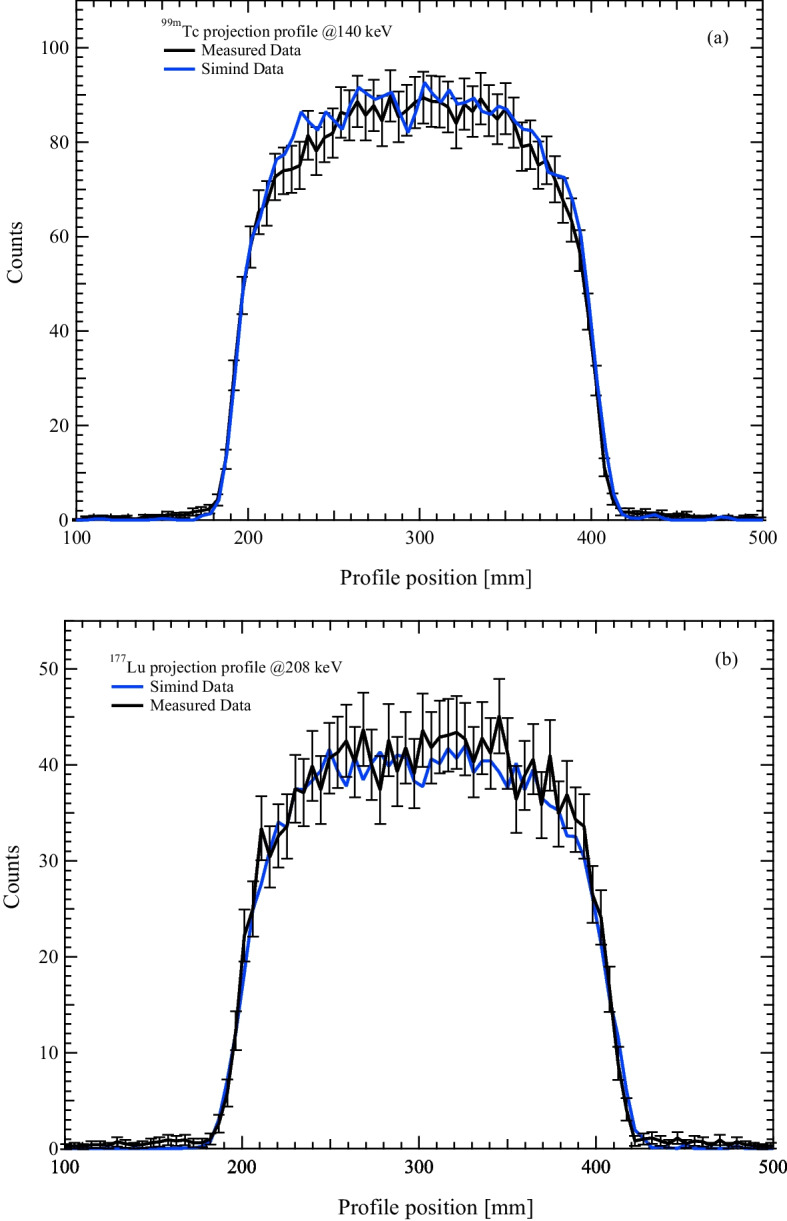
Comparison of experimental and simulated projection profiles. Plots of experimentally measured and Monte Carlo simulated profile of uniform cylinder filled with **a** $^{99m}$Tc and **b** $^{177}$Lu

**Fig. 5:**
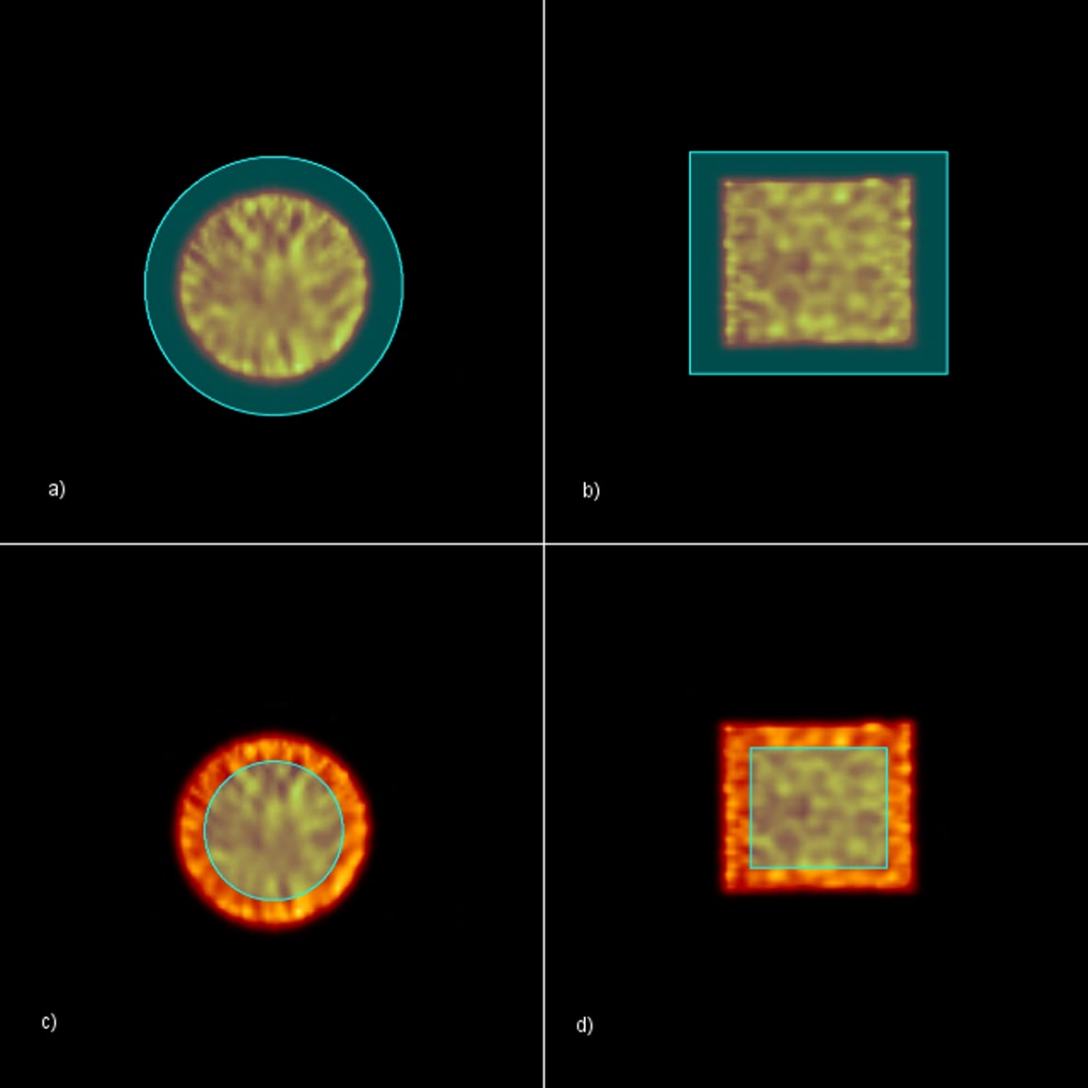
Volume of interest. SPECT reconstructed transverse **a** and sagittal **b** slices of the Jaszczak phantom togheter with type 1 VOI. SPECT reconstructed transverse **c** and sagittal **d** slices of the Jaszczak phantom togheter with type 2 VOI

**Fig. 6:**
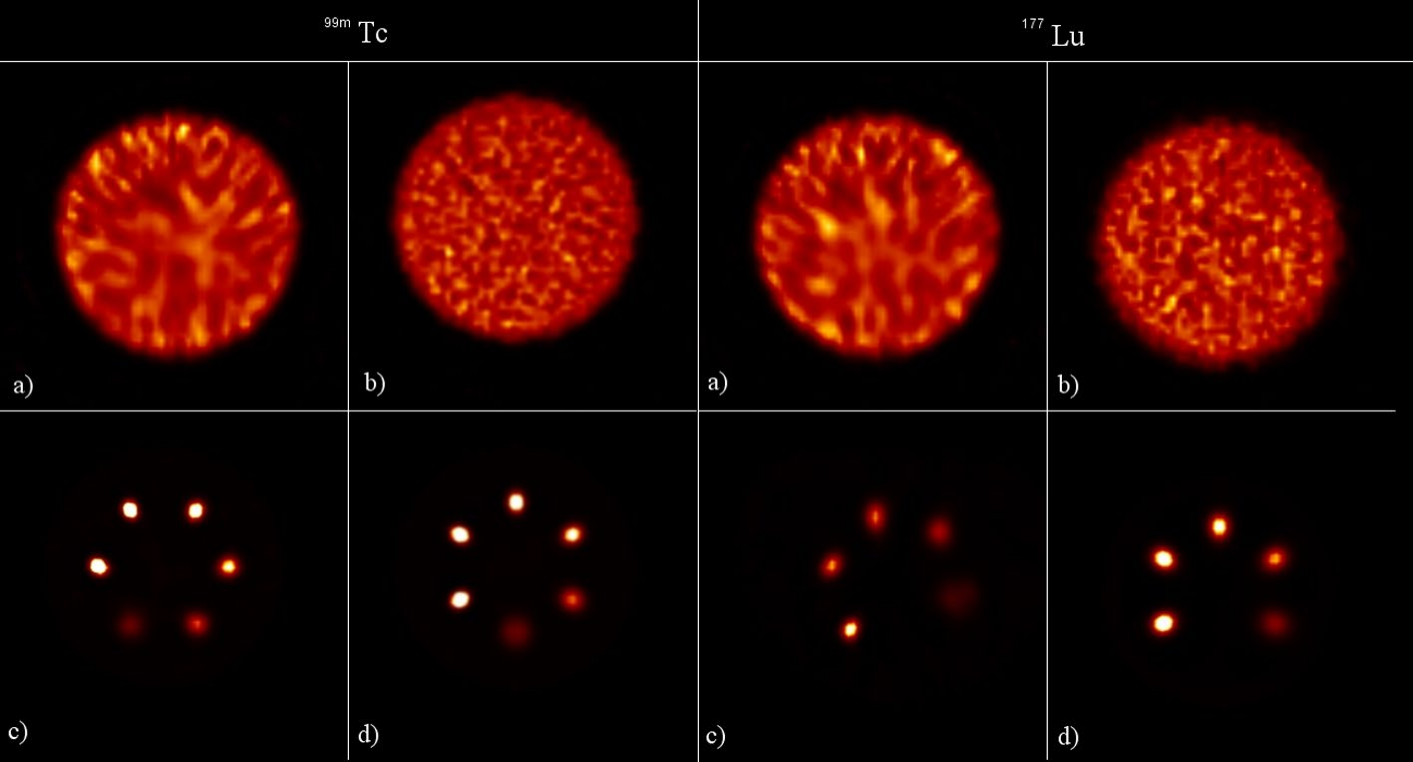
SPECT reconstructed transverse slices of the Jaszczak phantom without spheres **a** experimental and **b** simulated, with spheres **c** experimental and **d** simulated. On the left $^{99m}$Tc, on the right $^{177}$Lu

The first step was to compare the measured and simulated projection profiles of a uniform filled cylinder with $^{99m}$Tc or $^{177}$Lu. The horizontal profiles were obtained by drawing a line on a cylinder projection at three different positions and calculating the mean value for each profile coordinate. Figure 4a shows the result for $^{99m}$Tc, while the Fig. 4b shows the results for $^{177}$Lu. The error bar associated with each measured profile position was calculated as the square root of the counts in that position. Symbia Intevo CF has been evaluated for all the uniformity phantom acquisitions performed. Two cylindrical VOIs were used for CF evaluation: the type 1 VOI had linear dimensions 30% larger than those of the Jaszczak phantom but with the same geometrical centre, while the type 2 VOI had linear dimensions 30% smaller than those of the Jaszczak phantom but with the same geometrical centre. Figures 5a and 5b show transverse and coronal slices of the reconstructed Jaszczak phantom, respectively, together with VOI type 1. Figures 5c and 5d show transverse and coronal slices of the reconstructed Jaszczak phantom, respectively, together with VOI type 2. Figure 6 shows the transverse slices of the reconstructed Jaszczak phantom, without and with spheres, both for $^{99m}$Tc and $^{177}$Lu.

The errors associated with experimental results include:

- Errors in the evaluation of phantom volume;
- Error in activity evaluation through the calibrator (standard deviation of the measurement of the syringe samples, but also the systematic error of the calibrator itself);
- The standard deviation of the VOIs volume values.

The $^{99m}$Tc calculated CF value for the type 1 VOI was 110.1 ± 5.5 cps/MBq for experimental data while for SIMIND data the calculated value was 107.3 ± 0.3 cps/MBq. The CF values calculated for the type 2 VOI were 111.8 ± 5.6 cps/MBq and 113.8 ± 0.5 cps/MBq respectively for experimental and simulated data.

**Fig. 7:**
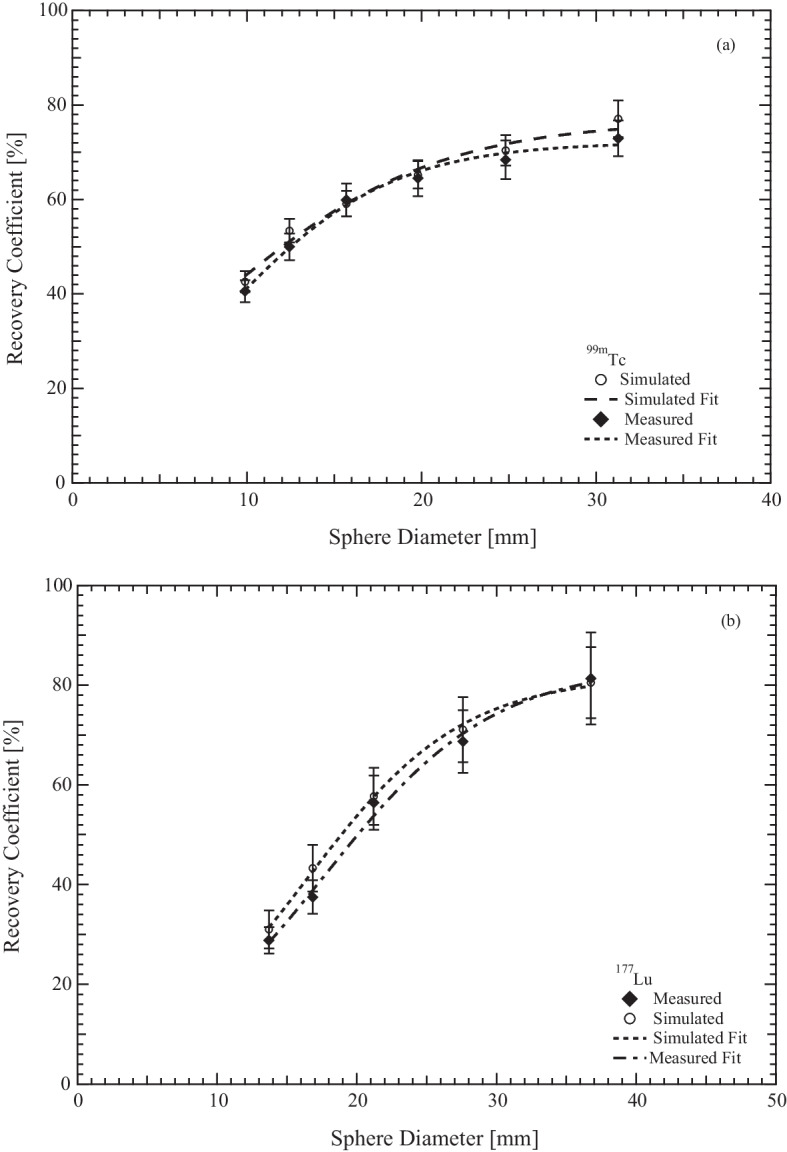
RC for $^{{\textbf {99m}}}$**Tc.** Plots of recovery coefficients (RC) as function of sphere diameter filled with **a** $^{99m}$Tc and **b** $^{177}$Lu

In figure 7a, the RC experimental values for $^{99m}$Tc are compared with the RC values obtained by using Monte Carlo simulation and reconstructed by CASToR software. To partially compensates for the CDR, a 2D Gaussian distribution has been used in the reconstruction process of the simulated data. The Gaussian FWHM has been chosen calculating the average distance of radioactive distribution inside the Jaszczak phantom from the collimator face, and selecting the corresponding FWHM value from Fig. 2a. Jentzen et al. [19] suggest to fit the RC data with a sigmoid curve function of sphere diameter D

3 $$\begin{aligned} RC = \frac{a}{1+b \cdot e^{-c \cdot D}} \end{aligned}$$

and the fitting curve is the dashed line on the plot. The $\chi ^2_r$ value of fit procedure was 0.9.

In order to obtain the CF factor for Lutetium, procedures followed for $^{99m}$Tc was repeated, resulting in a CF = 18.3 ± 1.0 cps/MBq for type 1 VOI, while the CF = 18.6 ± 1.0 cps/MBq for type 2 VOI.

The same uniformity phantom acquisitions have been repeated with SIMIND, in order to find CF for Lutetium. It is important to underline that CASToR can reconstruct studies taking into account only one peak at a time. So, two different values for CF (one for the peak at 113 keV and one for the peak at 208 keV) have been obtained. Usually, only the 208 keV peak is routinely used for dosimetry studies because of the low scattering/down scattering contribution, but the patient acquisition protocol for $^{177}$Lu used in Ferrara Hospital collects and reconstructs the data of both 113 keV and 208 keV peaks. In this case the CF was calculated as 20.4 ± 0.7 cps/MBq for the type 1 VOI and 21.4 ± 1.3 cps/MBq for the type 2 VOI. The RC coefficients have been evaluated from 1.4 ml to 26 ml, using spheres filled with Lutetium and the experimental and simulated data are in figure 7b with the fitting curve as dashed line. The $\chi ^2_r$ value of fit procedure was 0.17.

## Discussion

### Planar system resolution

The FWHM and FWTM values of the system spatial resolution at a distance of 10 cm for the experimental and simulated capillary planar images show a good agreement. The FWHM percentage difference is of 6% for $^{99m}$Tc, while the FWHM percentage differences are 3.4% for 113 keV peak and -5% for 208 keV peak of $^{177}$Lu. The results in this study correspond to the findings by Toossi et al. [9] for $^{99m}$Tc. They reported values of 8.4 ± 0.1 mm and 7.8 ± 0.1 mm for measured and simulated FWHM, respectively. For the 208 keV peak of $^{177}$Lu, Ramonaheng et al. [10] reported a value of 11.5 ± 0.35 mm for measured value, in agreement with the findings in this study.

### Planar system sensitivity

**Fig. 8:**
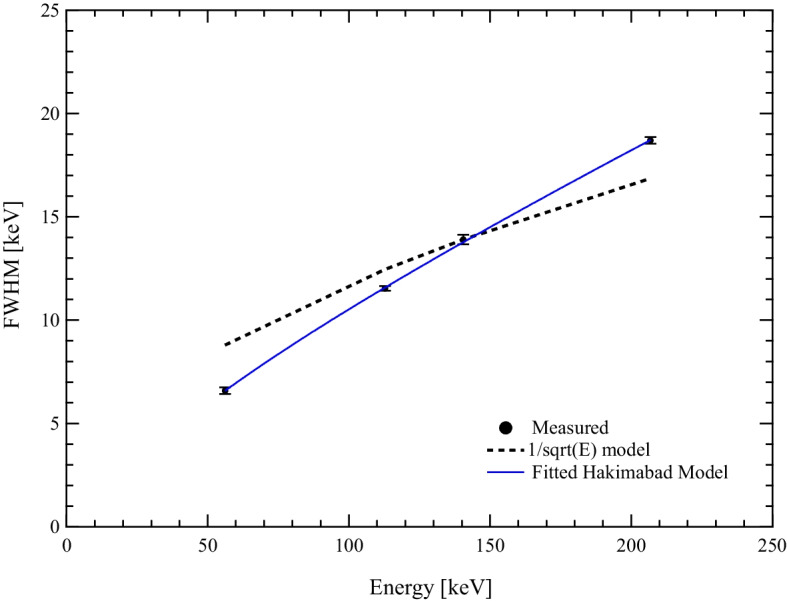
Gamma camera energy resolution. Comparison between the measured and calculated gamma camera energy resolution using two models

The results obtained for the system sensitivity acquired in planar imaging show an excellent agreement with the simulated data except for the 208 keV peak of $^{177}$Lu. Here, the value calculated using the MC simulation is 13.6% higher than the experimentally measured value. A possible explanation of this difference is how the energy resolution is modelled in SIMIND [20]. The energy resolution for the 140.5 keV peak of $^{99m}$Tc, and the 56.1 keV, 113 keV and 208 keV peaks of $^{177}$Lu have been calculated from the energy spectrum acquired during the sensitivity measurement. Figure 8 shows the measured energy resolution as a function of peak energy, the curve calculated by the $1/\sqrt{(}E)$ model and the curve fitting with the Hakimabad model [21] as proposed by Morphis [20]. Comparing the energy resolution values for the $^{177}$Lu predicted by the model $1/\sqrt{(}E)$ with the experimental values (Fig 6), the simulated energy resolution is 1% more for the 113 keV and 1% less for the 208 keV peak in comparison with the experimental values. This difference in energy resolution will result in less simulated counts for the 113 keV peak and more counts for the 208 keV peak when compared to the experimental counts, which explains the sensitivity difference seen in Table 8. To verify this effect, a $^{177}$Lu sensitivity study (activity in a petri dish) was simulated using two different fixed energy resolution values. For the first simulation the measured energy resolution at 113 keV was used and for the second simulation the 208 keV measured energy resolution value. Analysing the simulated spectra and calculating the area under the two peaks for the two simulated studies, resulted in a count increase of 4% for the 113 keV PW and a count decrease of 5% for the 208 keV PW. This will effectively change the results in table 8 for the $^{177}$Lu simulated sensitivity from 9.7 cps/MBq to 10.1 cps/MBq for the 113 keV peak and from 10.9 cps/MBq to 10.4 cps/MBq for the 208 keV peak.

The remaining difference respect to the measured values could be due to the uncertainty on $^{177}$Lu activity.

Ramonaheng et al. [10] have reported for the 208 keV peak of $^{177}$Lu the experimental and MC (SIMIND) simulated value of 10.0 ± 0.3 cps/MBq and 10.3 cps/MBq, respectively. Both values compare well with the results in this study. Finally, since the sensitivity in SPECT imaging decreases as activity increases, it is necessary to assess when the dead time correction is needed in order to apply it so to have a correct quantification of the volume activity (Table 9).

**Table 9:** Experimental and simulated CF values for $^{99m}$Tc and $^{177}$Lu obtained from acquisitions with the Jaszczak phantom

Parameter	Method	Radioisotope	Main peak [keV]	Experimental	Simulated
CF [cps/MBq]	Ellipsoid	Tc-99m	140.5	110.1 ± 5.5	107.3 ±0.3
CF [cps/MBq]	Ellipsoid	Lu-177	113.0+208.0	18.3 ± 1.0	20.4 ± 0.7

### CF and RC for $^{99m}$Tc and $^{177}$Lu

The parameters acquired in tomographic mode, CF and RC, require a further step to be calculated: tomographic reconstruction using appropriate software. Usually, an iterative reconstruction algorithm is used for SPECT reconstruction that allows the inclusion of the following three contributions: the collimator response function, scatter contribution, the attenuation in tissue. Thus, the parameters calculated from the reconstructed images depend on how correct the estimate of the three contributions is.

As it was observed by Zhao et al. [22] CF value calculated by a tomographic study is higher than that determined by planar scan. Additionally, the same authors stated that the scatter correction evaluated by DEW or TEW method could introduce error in the calculation of CF value, and the error depends on source distribution inside the phantom or the patient. Peters et al. [23] reported an experimental CF value for $^{99m}$Tc of 112  cps/MBq for a Symbia Intevo 6 gamma camera, that compares well with our result of 110.1 ± 5.5 cps/MBq.

To estimate the RC curve using the simulated data with SIMIND, we have calculated an average gamma camera spatial resolution averaged over the activity distribution. In fact, the software CASTOR at the moment allows inclusion of a Gaussian collimator response function not dependent on the distance. For $^{99m}$Tc the selected FWHM was 11.1 mm, while for $^{177}$Lu the average values were 21.4 mm and 22.5 mm for 113 and 208 peak, respectively. Simulated RC for $^{99m}$Tc agrees well with the experimental value, but the maximum value of RC is 71% for a sphere diameter of 31.6 mm. Zeintl et al. [7] reported a value of 80% for the same sphere diameter. By increasing the number of iterations, it is possible to obtain better RC values. Simulated RC for $^{177}$Lu are in excellent agreement with the experimental one, the maximum value of RC being 80% for a sphere diameter of 37 mm. Sanders et al. [24] report a maximum RC value of 78% for a sphere diameter of 31.6 mm. Zeintl et al. [7] have analysed the change in RC values as a function of background-sphere ratio for 16 ml sphere. The authors reported the RC value changes starting from 76% to 78% if the background-sphere ratio is less than 10%. If this result were still valid for the smaller spheres, the curves calculated in this study would not be different from those obtained with a constant background-sphere ratio less than 10%.

## Conclusions

In this study, we have verified a modelled Symbia Intevo Excel by comparing the system resolution and the system planar sensitivity both for $^{99m}$Tc and $^{177}$Lu. We validated the CF and the RC derived from a MC modelled gamma camera by comparing results from physical measurements to SIMIND simulations for the isotope-collimator combinations $^{99m}$Tc-LEHR, $^{177}$Lu-MELP. Results show that appropriate corrections like attenuation, scatter and collimator detector response are essential when activity quantification is needed. A shortcoming of this study is the evaluation of the effect of the number of iterations and subsets on quantitative SPECT imaging, and it is suggested that it should be done in a follow-up study. Overall, it has been shown that SIMIND is a useful tool to simulate gamma cameras, using several radionuclides for different purpose both in the diagnostic and therapeutic fields.

## Data Availability

Not applicable
